# Prevalence of Doctor-Diagnosed Arthritis and Arthritis-Attributable Activity Limitation — United States, 2010–2012

**Published:** 2013-11-08

**Authors:** Kamil E. Barbour, Charles G. Helmick, Kristina A. Theis, Louise B. Murphy, Jennifer M. Hootman, Teresa J. Brady, Yiling J. Cheng

**Affiliations:** Div of Population Health; Div of Diabetes Translation, National Center for Chronic Disease Prevention and Health Promotion, CDC

Arthritis is the most common cause of disability among U.S. adults and is particularly common among persons with multiple chronic conditions (1). In 2003, arthritis in the United States resulted in an estimated $128 billion in medical-care costs and lost earnings (2). To update previous U.S. estimates (3) of the prevalence of doctor-diagnosed arthritis and arthritis-attributable activity limitation (AAAL), CDC analyzed 2010–2012 data from the National Health Interview Survey (NHIS). This report summarizes the results of that analysis, which found that 52.5 million (22.7%) of adults aged ≥18 years had self-reported doctor-diagnosed arthritis, and 22.7 million (9.8%, or 43.2% of those with arthritis) reported AAAL, matching and exceeding previous projected increases, respectively (4). Among persons with heart disease, diabetes, and obesity, the prevalences of doctor-diagnosed arthritis were 49.0%, 47.3%, and 31.2%, respectively; the prevalences of AAAL among persons with these specific conditions were 26.8%, 25.7%, and 15.2%, respectively. Greater use of evidence-based interventions, such as chronic disease self-management education and physical activity interventions that have been proven to reduce pain and improve quality-of-life among adults with chronic diseases might help reduce the personal and societal burden of arthritis.

NHIS is an annual, nationally representative, in-person interview survey of the health status and behaviors of the non-institutionalized civilian U.S. population. In each household identified, one adult was randomly selected to complete the “sample adult” questionnaire.* Participants were categorized into five racial/ethnic groups: Hispanic, white, black, Asian, and other race. Persons identified as Hispanic might be of any race. Persons identified as white, black, Asian, or other race all were non-Hispanic. Sampling weights were applied to account for household nonresponse and oversampling of blacks, Hispanics, and Asians. Poststratification adjustments were applied by NCHS using 2000 U.S. Census estimates for the years 2010–2011, and 2010 U.S. Census estimates for 2012. For this analysis, NHIS data from 2010, 2011, and 2012 were combined, and annualized prevalence estimates were calculated overall and stratified by selected characteristics (i.e., sex, age group, race/ethnicity, education level, employment status, body mass index (BMI) category,† physical activity level,§ self-rated health, doctor-diagnosed heart disease,¶ and doctor-diagnosed diabetes). Unweighted sample sizes and final response rates were 27,157 (60.8%) in 2010, 33,014 (66.3%) in 2011; and 34,525 (61.2%) in 2012.

Adults were defined as having doctor-diagnosed arthritis if they answered “yes” to “Have you ever been told by a doctor or other health professional that you have some form of arthritis, rheumatoid arthritis, gout, lupus, or fibromyalgia?” Those who responded “yes” were also asked, “Are you now limited in any way in any of your usual activities because of arthritis or joint symptoms?” Those responding “yes” to both questions were categorized as having AAAL. Prevalence of AAAL was estimated for the overall adult U.S. population and for adults with arthritis.

All analyses were weighted to account for the complex multistage sampling design. Unadjusted prevalence estimates for arthritis and AAAL describe the absolute population burden. Age-adjusted prevalence estimates (standardized to the projected 2000 U.S. standard population) describe relative population burden among various analytic subgroups. For all comparisons, differences were considered statistically significant if the 95% confidence intervals of the age-adjusted estimates did not overlap.

An estimated 22.7% (52.5 million) of U.S. adults reported doctor-diagnosed arthritis, including 49.7% of adults aged ≥65 years. High arthritis prevalence was observed among adults with heart disease (49.0%) and diabetes (47.3%). In age-adjusted analyses, arthritis prevalence was significantly higher among women than men, among whites and blacks compared with Hispanics and Asians, among those with less education, those who were obese or overweight, and those not meeting physical activity recommendations. Arthritis prevalence (age-adjusted) also was higher among those who were unable to work or were disabled (29.0%) compared with those who were employed (20.9%), and higher among those with self-reported fair or poor health (40.7%) compared with those reporting excellent or very good health (15.8%) (Table).

Among adults with doctor-diagnosed arthritis, the unadjusted overall prevalence of AAAL was 43.2% (22.7 million persons or 9.8% of the overall population). The highest AAAL prevalence among adults with arthritis was for those who reported fair or poor health (71.8%), were unable to work or disabled (61.4%), were physically inactive (56.5%), had less than a high school diploma (55.4%), had heart disease (54.6%), or had diabetes (54.4%). These patterns persisted after age-adjustment. Age-adjusted AAAL prevalence among adults with doctor-diagnosed arthritis was higher for Hispanics compared with whites, even though Hispanics’ age-adjusted prevalence of arthritis in the general population was lower, suggesting greater average severity of arthritis among Hispanics (Table).

In unadjusted analyses, adults with heart disease (11.5%) and diabetes (9.0%), 49.0% and 47.3% had arthritis, respectively, and more than a quarter for each condition had AAAL. Among obese adults (28.2%), 31.2% had arthritis and 15.2% had AAAL (Table).

What is already known on this topic?

Arthritis is the most common cause of disability among U.S. adults, resulting in annual costs estimated at $128 billion in 2003, and is particularly common among persons with multiple chronic conditions.

What is added by this report?

During 2010–2012, an estimated 22.7% of adults had self-reported doctor-diagnosed arthritis, and 43.2% of those with arthritis reported arthritis-attributable activity limitations (AAAL). Approximately half of all adults with heart disease or diabetes had arthritis, and one fourth of adults with either condition and arthritis had AAAL. Approximately one third of adults who were obese also had arthritis, and 15% of those adults had AAAL.

What are the implications for public health practice?

Health-care providers and public health practitioners can address both arthritis and other chronic conditions by prioritizing self-management education and appropriate physical activity as effective ways to improve health outcomes (e.g., reducing pain and increasing function and quality-of-life).

## Editorial Note

During 2010–2012, an estimated 52.5 million (22.7%) of adults in the United States reported doctor-diagnosed arthritis, and 22.7 million (9.8%) reported AAAL (43.2% of those with arthritis). These estimates represent net increases of 0.87 million adults with arthritis per year and 0.53 million adults with AAAL per year since the 2007–2009 estimates of 49.9 million with arthritis and 21.1 million with AAAL (3). These increases can be attributed, in part, to the aging of the U.S. population. The arthritis estimate is consistent with an earlier projection and suggests that projections of 55.7 million adults with arthritis by 2015 and 67 million by 2030 (4) are reasonable. For AAAL, the estimate exceeds the earlier projection of 22 million adults with AAAL by 2020 and, therefore, might exceed the 25 million projected for 2030 (4).

Arthritis and AAAL create a substantial personal and societal burden in the United States. Arthritis and AAAL prevalences were greater in the same age, sex, race/ethnicity, and education subgroups as seen previously (3), and exceptionally high among those who were unable to work or were disabled and those with fair or poor health, even when adjusted for age. About half of all adults with heart disease or diabetes had arthritis, and more than a quarter of adults with either condition and arthritis had AAAL; almost one third of adults who were obese also had arthritis, and more than 15% of these adults had AAAL. The high prevalence of arthritis among adults with these conditions in the general population is consistent with the results of a previous study on co-occurrence of chronic diseases among adults aged ≥25 years who participated in NHIS, in which arthritis was among the most common comorbidities (5). The negative effects of combinations of arthritis and other chronic conditions are suggested by the AAAL findings in this analysis, along with studies identifying arthritis as associated with greater physical inactivity for adults with multiple chronic conditions (6–8).

The findings in this report are subject to at least four limitations. First, doctor-diagnosed arthritis was self-reported and not confirmed by a health-care professional; however, this case definition has been shown to be sufficiently sensitive for public health surveillance (9). Second, because NHIS is a cross-sectional survey, a causal relationship between risk factors (i.e., obesity or physical activity) and arthritis and AAAL could not be established. Nonetheless, obesity is a factor that increases risk for osteoarthritis; a prospective study with 10 years of follow-up found that obese adults were more than twice as likely to develop knee and hand osteoarthritis (10). Third, social desirability bias might play a role in some self-report characteristics, with underreporting of weight, overreporting of height, and overreporting of leisure-time physical activity. Finally, because response rates ranged from 60.8% to 66.3% the findings might be subject to selection bias, although the application of sampling weights is expected to considerably reduce nonresponse bias.

A current U.S. Department of Health and Human Service initiative** addresses the burden of multiple chronic conditions, which now affect one in four adults and are increasingly common with the aging of the population. The findings in this report indicate that arthritis commonly co-occurs with obesity as well as heart disease and diabetes, and that high prevalence of AAAL is found among adults with both arthritis and one of these chronic conditions. CDC is promoting greater coordination with state health departments to address these chronic disease comorbidity concerns.†† An opportunity for collaboration is the dissemination of information regarding evidence-based self-management education and physical activity interventions§§ that have been proven to reduce pain and improve function, mood, confidence to manage health, and quality of life. The physical activity interventions recommended are appropriate exercise regimens intended to reduce activity limitations among adults with arthritis and assuage concerns over aggravating the condition.¶¶ CDC currently funds arthritis programs in 12 states to disseminate information and implement programs in local communities.*** Given the high prevalence of arthritis and AAAL among adults with certain chronic conditions and the arthritis-specific barriers to activity (6–8), health-care providers and public health practitioners can address both arthritis and these other chronic conditions by prioritizing self-management education and appropriate physical activity as an effective way to improve health outcomes.

## Figures and Tables

**TABLE:** Unadjusted and age-adjusted* annualized prevalence of doctor-diagnosed arthritis and arthritis-attributable activity limitation (AAAL)† among adults aged ≥18 years, and prevalence of AAAL among those with doctor-diagnosed arthritis, by selected characteristics — National Health Interview Survey, United States, 2010–2012

Characteristic	%	Prevalence in the adult population								Prevalence of AAAL among adults with doctor-diagnosed arthritis			

		Doctor-diagnosed arthritis				AAAL							
		
		Unadjusted		Adjusted		Unadjusted		Adjusted		Unadjusted		Adjusted	
					
		%	(95% CI)	%	(95% CI)	%	(95% CI)	%	(95% CI)	%	(95% CI)	%	(95% CI)
**Overall**	**—**	**22.7**	**(22.3–23.0)**	**21.4**	**(21.1–21.7)**	**9.8**	**(9.5–10.1)**	**9.2**	**(9.0–9.4)**	**43.2**	**(42.4–44.1)**	**40.7**	**(39.5–41.9)**
**Age group (yrs)**													
18–44	47.8	7.3	(7.0–7.6)	—	—	2.7	(2.6–2.9)	—	—	37.5	(35.4–39.7)	—	—
45–64	34.9	30.3	(29.8–30.9)	—	—	13.4	(12.9–13.9)	—	—	44.2	(42.9–45.5)	—	—
≥65	17.3	49.7	(48.7–50.6)	—	—	22.0	(21.3–22.8)	—	—	44.4	(43.2–45.6)	—	—
**Sex**													
Men	48.3	19.1	(18.6–19.7)	18.6	(18.2–19.0)	8.0	(7.7–8.4)	7.8	(7.5–8.1)	41.9	(40.5–43.3)	39.2	(37.2–41.3)
Women	51.7	26.0	(25.5–26.5)	23.9	(23.5–24.3)	11.5	(11.1–11.8)	10.5	(10.2–10.8)	44.2	(43.2–45.2)	41.7	(40.2–43.2)
**Race/Ethnicity** §													
White	68.0	25.9	(25.5–26.4)	22.9	(22.5–23.3)	10.8	(10.5–11.1)	9.5	(9.2–9.7)	41.7	(40.7–42.6)	39.3	(37.8–40.8)
Black	11.9	21.3	(20.3–22.2)	22.4	(21.6–23.2)	10.5	(9.8–11.2)	11.0	(10.4–11.7)	49.3	(47.2–51.4)	47.0	(44.4–49.7)
Hispanic	14.3	12.1	(11.5–12.7)	15.9	(15.2–16.6)	5.9	(5.5–6.3)	8.0	(7.5–8.6)	48.8	(46.3–51.4)	44.8	(41.5–48.2)
Asian	4.9	11.0	(10.0–12.0)	12.1	(11.2–13.1)	4.5	(3.9–5.2)	5.1	(4.5–5.8)	41.1	(36.6–45.7)	30.4	(25.2–36.2)
Other races	0.8	27.0	(23.2–31.2)	27.9	(24.3–31.9)	16.3	(13.3–19.8)	17.0	(14.1–20.4)	60.1	(52.4–67.4)	55.8	(45.7. 65.4)
**Education level**													
<High school diploma	14.2	25.7	(24.8–26.6)	21.9	(21.2–22.7)	14.2	(13.5–15.0)	12.2	(11.5–12.8)	55.4	(53.6–57.3)	53.9	(50.4–57.3)
High school diploma	26.6	25.6	(25.0–26.3)	23.0	(22.4–23.5)	11.4	(11.0–11.9)	10.2	(9.8–10.6)	44.6	(43.1–46.0)	42.2	(40.0–44.4)
At least some college	31.0	22.7	(22.1–23.4)	23.3	(22.8–23.8)	9.6	(9.2–10.1)	9.9	(9.5–10.3)	42.4	(40.9–43.8)	40.6	(38.6–42.6)
Completed college or greater	28.1	18.3	(17.7–18.9)	17.8	(17.2–18.3)	6.2	(5.8–6.5)	6.0	(5.7–6.3)	33.7	(32.1–35.3)	30.4	(28.4–32.4)
**Body mass index (BMI)** ¶													
Under/Normal weight	37.1	15.9	(15.4–16.4)	16.3	(15.9–16.7)	6.3	(6.0–6.6)	6.5	(6.2–6.8)	39.8	(38.4–41.3)	38.2	(35.8–40.7)
Overweight	34.7	22.6	(22.0–23.2)	20.3	(19.8–20.8)	8.8	(8.4–9.2)	7.9	(7.5–8.2)	38.9	(37.6–40.2)	37.2	(35.3–39.2)
Obese	28.2	31.2	(30.5–32.0)	28.9	(28.3–29.5)	15.2	(14.7–15.7)	14.0	(13.5–14.5)	48.6	(47.3–49.9)	44.8	(42.9–46.6)
**Physical activity** **													
Meeting recommendations	48.3	17.4	(17.0–17.8)	18.6	(18.2–19.0)	5.3	(5.0–5.5)	5.6	(5.4–5.9)	30.2	(29.0–31.5)	29.3	(27.7–31.0)
Insufficient activity	20.0	25.3	(24.6–26.1)	23.3	(22.6–24.0)	10.3	(9.8–10.8)	9.4	(8.9–9.9)	40.6	(38.8–42.4)	38.9	(36.6–41.3)
Inactive	31.6	28.9	(28.2–29.7)	24.0	(23.4–24.6)	16.3	(15.8–16.9)	13.5	(13.0–13.9)	56.5	(55.2–57.7)	54.8	(52.7–56.8)
**Employment status**													
Employed/Self–employed	64.9	18.8	(18.4–19.2)	20.9	(20.5–21.4)	7.9	(7.7–8.2)	9.0	(8.7–9.4)	42.3	(41.1–43.6)	40.0	(38.5–41.5)
Unemployed	7.2	14.0	(12.8–15.2)	19.0	(17.3–20.8)	6.1	(5.5–6.9)	8.4	(7.2–9.8)	43.9	(40.0–47.9)	43.2	(38.8–47.8)
Unable to work/Disabled	1.5	29.5	(26.7–32.5)	29.0	(26.3–31.8)	18.1	(15.8–20.7)	17.5	(15.1–20.2)	61.4	(55.7–66.9)	61.7	(54.2–68.7)
Other††	26.5	34.2	(33.4–35.1)	21.4	(20.8–22.1)	14.9	(14.4–15.4)	9.2	(8.8–9.6)	43.4	(42.2–44.6)	41.0	(37.7–44.5)
**Self–rated health**													
Very good/Excellent	60.3	14.4	(14.0–14.8)	15.8	(15.4–16.1)	3.4	(3.2–3.6)	3.7	(3.5–5.9)	23.5	(22.4–24.6)	22.3	(20.8–23.9)
Good	26.7	28.0	(27.3–28.7)	24.4	(23.8–25.0)	11.6	(11.1–12.0)	10.0	(9.6–10.4)	41.3	(40.0–42.7)	39.9	(37.7–42.1)
Fair/Poor	13.0	50.1	(49.1–51.2)	40.7	(39.5–41.9)	35.9	(34.9–36.9)	28.8	(27.8–29.9)	71.8	(70.5–73.0)	69.8	(67.8–71.8)
**Heart disease** §§													
Yes	11.5	49.0	(47.9–50.2)	35.4	(34.0–36.8)	26.8	(25.8–27.7)	19.4	(18.4–20.4)	54.6	(53.0–56.1)	54.0	(50.6–57.3)
No	88.5	19.2	(18.9–19.6)	19.6	(19.3–19.9)	7.6	(7.3–7.8)	7.7	(7.5–7.9)	39.4	(38.5–40.3)	37.8	(36.6–39.1)
**Diabetes** ¶¶													
Yes	9.0	47.3	(46.0–48.6)	34.0	(32.5–35.7)	25.7	(24.6–26.9)	18.8	(17.6–20.1)	54.4	(52.4–56.3)	55.9	(52.0–59.7)
No	91.0	20.2	(19.9–20.6)	20.2	(19.9–20.5)	8.2	(8.0–8.5)	8.2	(8.0–8.4)	40.6	(39.7–41.5)	38.7	(37.4–39.9)

**Abbreviation:** CI = confidence interval.

* Age adjusted to the 2000 U.S. projected adult population, using three age groups: 18–44, 45–64, and ≥65 years.

† Doctor–diagnosed arthritis was defined as an affirmative response to the question, “Have you ever been told by a doctor or other health professional that you have some form of arthritis, rheumatoid arthritis, gout, lupus, or fibromyalgia? Those who answered “yes” were asked, “Are you now limited in any way in any of your usual activities because of arthritis or joint symptoms?” Persons responding “yes” to both questions were defined as having AAAL.

§ Race/ethnicity categories are mutually exclusive. Persons identified as Hispanic might be of any race. Persons identified as white, black, Asian, or other race all were non-Hispanic.

¶ BMI = self–reported weight (kg) / (height [m]) ^2^. Categorized as follows: underweight/normal weight (<25.0), overweight (25.0 to <30.0), obese (≥30.0).

** Determined from responses to six questions regarding frequency and duration of participation in leisure–time activities of moderate or vigorous intensity and categorized according to the U.S. Department of Health and Human Services *2008 Physical Activity Guidelines for Americans*. Total minutes (moderate to vigorous) of physical activity per week were categorized as follows: meeting recommendations (≥150 min per week), insufficient activity (1–149 min), and inactive (0 min).

†† Students, volunteers, homemakers, and retirees.

§§ Adults were considered to have doctor–diagnosed heart disease if they answered “yes” to any of the following four questions: “Have you ever been told by a doctor or other health professional that you had coronary heart disease? Angina, also called angina pectoris? A heart attack (also called myocardial infarction)? Any kind of heart condition or heart disease (other than the ones I just asked about)?”

¶¶ Adults were considered to have doctor–diagnosed diabetes disease if they answered “yes” to “Have you ever been told by a doctor or health professional that you have diabetes or sugar diabetes?”

## Footnotes

* Survey description documents are available at http://www.cdc.gov/nchs/nhis/quest_data_related_1997_forward.htm.

† BMI = weight (kg) / (height [m])^2^. Categorized as follows: underweight/normal weight (<25.0), overweight (25.0 to <30.0), obese (≥30.0).

§ Determined from responses to six questions regarding frequency and duration of participation in leisure-time activities of moderate or vigorous intensity and categorized according to the U.S. Department of Health and Human Services *2008 Physical Activity Guidelines for Americans*. Total minutes (moderate to vigorous) of physical activity per week were categorized as follows: meeting recommendations (≥150 min per week), insufficient activity (1–149 min), and inactive (0 min).

¶ Adults were considered to have doctor-diagnosed heart disease if they answered “yes” to any of the following four questions: “Have you ever been told by a doctor or other health professional that you had coronary heart disease? Angina, also called angina pectoris? A heart attack (also called myocardial infarction? Any kind of heart condition or heart disease (other than the ones I just asked about)?”

** Available at http://www.hhs.gov/ash/initiatives/mcc.

†† Available at http://www.cdc.gov/chronicdisease/about/statepubhealthactions-prevcd.htm.

§§ Additional information available at http://www.cdc.gov/arthritis/interventions.htm.

¶¶ Additional information available at http://www.cdc.gov/arthritis/pa_overview.htm.

*** Available at http://www.cdc.gov/arthritis/state_programs/programs.
